# Marine Microbial-Derived Resource Exploration: Uncovering the Hidden Potential of Marine Carotenoids

**DOI:** 10.3390/md20060352

**Published:** 2022-05-26

**Authors:** Ray Steven, Zalfa Humaira, Yosua Natanael, Fenny M. Dwivany, Joko P. Trinugroho, Ari Dwijayanti, Tati Kristianti, Trina Ekawati Tallei, Talha Bin Emran, Heewon Jeon, Fahad A. Alhumaydhi, Ocky Karna Radjasa, Bonglee Kim

**Affiliations:** 1 Institut Teknologi Bandung, School of Life Sciences and Technology, Bandung 40132, Indonesia; ray.steven@students.itb.ac.id (R.S.); zalfahhumaira@students.itb.ac.id (Z.H.); yosua.nat@students.itb.ac.id (Y.N.); 2 Department of Life Sciences, Imperial College London, South Kensington Campus, London SW72AZ, UK; j.trinugroho16@imperial.ac.uk; 3 CNRS@CREATE Ltd., 1 Create Way, #08-01 Create Tower, Singapore 138602, Singapore; ari.dwijayanti@cnrsatcreate.sg; 4 Institut Pendidikan Indonesia, Garut 44151, Indonesia; tati@institutpendidikan.ac.id; 5 Department of Biology, Faculty of Mathematics and Natural Sciences, Sam Ratulangi University, Manado 95115, Indonesia; trina_tallei@unsrat.ac.id; 6 Department of Pharmacy, BGC Trust University Bangladesh, Chittagong 4381, Bangladesh; talhabmb@bgctub.ac.bd; 7 Department of Pharmacy, Faculty of Allied Health Sciences, Daffodil International University, Dhaka 1207, Bangladesh; 8 Department of Pathology, College of Korean Medicine, Kyung Hee University, 1-5 Hoegidong, Seoul 02447, Korea; titmal@khu.ac.kr; 9 Department of Medical Laboratories, College of Applied Medical Sciences, Qassim University, Buraydah 52571, Saudi Arabia; f.alhumaydhi@qu.edu.sa; 10 Oceanography Research Center, The Earth Sciences and Maritime Research Organization, National Research and Innovation Agency, North Jakarta 14430, Indonesia

**Keywords:** carotenoids, microorganisms, marine, biosynthetic gene cluster

## Abstract

Microbes in marine ecosystems are known to produce secondary metabolites. One of which are carotenoids, which have numerous industrial applications, hence their demand will continue to grow. This review highlights the recent research on natural carotenoids produced by marine microorganisms. We discuss the most recent screening approaches for discovering carotenoids, using in vitro methods such as culture-dependent and culture-independent screening, as well as in silico methods, using secondary metabolite Biosynthetic Gene Clusters (smBGCs), which involves the use of various rule-based and machine-learning-based bioinformatics tools. Following that, various carotenoids are addressed, along with their biological activities and metabolic processes involved in carotenoids biosynthesis. Finally, we cover the application of carotenoids in health and pharmaceutical industries, current carotenoids production system, and potential use of synthetic biology in carotenoids production.

## 1. Introduction

The ocean is a valuable ecosystem for discovering new natural products with a variety of applications, one of which is the search for microbes that produce secondary metabolites [1,2]. The differences in physical, chemical, and biological properties in each water column make the components of organisms that make up the oceans different. The structure of the bacterial community varies significantly with depth differences in the open ocean [3,4,5]. Coral reef ecosystems are classified as productive ecosystems due to their location in the photic zone, which is exposed to direct sunlight and supports a high biodiversity of macro- and microorganisms. The mucus layer of corals is rich in polysaccharides and is used as a growth medium by a variety of bacterial communities, including those that produce bioactive compounds [6,7]. Meanwhile, the deep sea is located in the aphotic zone with a depth of >200 m, which has extreme and unique ecological properties. The lack of sunlight, low temperature, high hydrostatic pressure, and oligotrophic environment all contribute to the deep sea being a habitat for adaptive microorganisms with distinctive metabolic characteristics from those found in the photic zone [8]. The abundance of literature discussing secondary metabolite exploration from coastal areas to the deep sea demonstrates tremendous and significant potential for the discovery of this resource (reviewed in ref. [9]).

**Figure 1:**
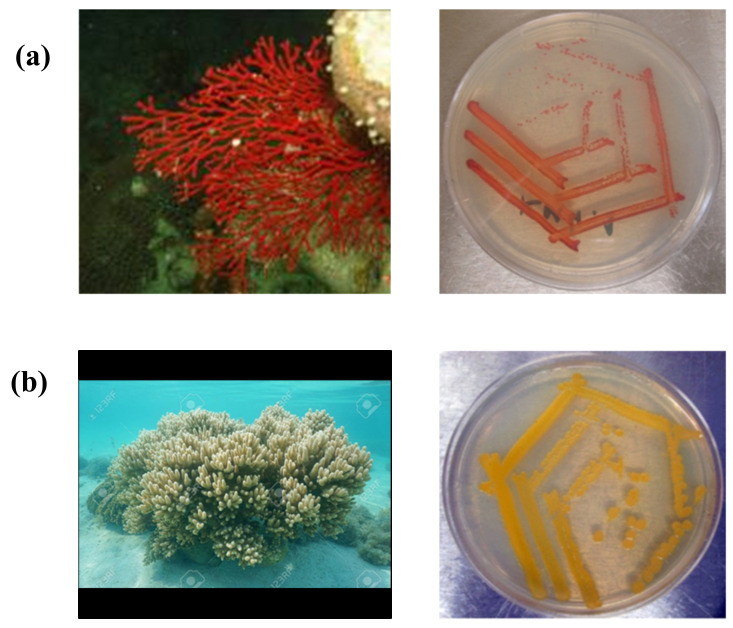
Corals with their carotenoid-producing bacteria. (**a**) *Leptogorgia* sp. with red-colored morphology and red-colored colony reflects red-colored carotenoid. (**b**) *Sinularia* sp. with yellow-colored morphology and yellow-colored colony reflects yellow-colored carotenoid.

Secondary metabolites are low-molecular-weight compounds that are not directly involved in an organism’s primary metabolic processes. They are, however, necessary for the long-term survival of these species [10]. At the end of 2016, approximately 28,500 secondary metabolites were discovered in marine organisms, including marine microorganisms (bacteria and fungi), porifera, cnidaria, marine algae, and marine phytoplankton (such as dinoflagellates and cyanobacteria) [11,12]. Numerous publications reported the discovery of new secondary metabolites, including 1490 compounds in 2017 [13], 1554 compounds in 2018 [14], and 1490 compounds in 2019 [15]. In general, these secondary metabolites can be grouped into polyketides (PKS), nonribosomal peptides (NRPs), hybrid PKS and NRPs (PKS/NRPs), terpenoids/isoprenoids, ribosomally synthesized and post-translationally modified peptides (RiPPs), and bisindoles [16]. Carotenoids are a class of secondary metabolites belonging to the terpenoids/isoprenoids [17]. Certain carotene-producing bacteria live with coral symbionts [18] (Figure 1) such as the Red Gorgonian (*Leptogorgia* sp.) and Softcorals (*Sinularia* sp.) [19].

Due to the important role of carotenoids in the industry, this review emphasizes the current knowledge and technologies on marine carotenoids types, function, screening methods, and its utilization in health and pharmaceutical industries. First, this review discusses the characteristics, biological function, and biosynthetic gene cluster of marine carotenoids. Second, this review discusses the various in vitro and in silico screening methods for marine carotenoid. Lastly, this review discusses the economic potential of marine carotenoids and its usage in the health and pharmaceutical industry.

## 2. Carotenoids

### 2.1. Characteristics and Biological Function of Carotenoids

Carotenoids are one of the isoprenoid derivatives with distinctive colors formed by six conjugated double bonds in their polyene chains, each double bond being either *trans* or *cis* and containing 40 carbon atoms (tetraterpenes) [20,21] and formed by head-to-head association [22]. Carotenoids are typically found in *trans* form in nature [23,24], but can be commercialized in *cis* form due to the influence of environmental factors such as temperature and light [25]. Carotenoids are also easily degraded under the influence of oxygen [24].

**Figure 2:**
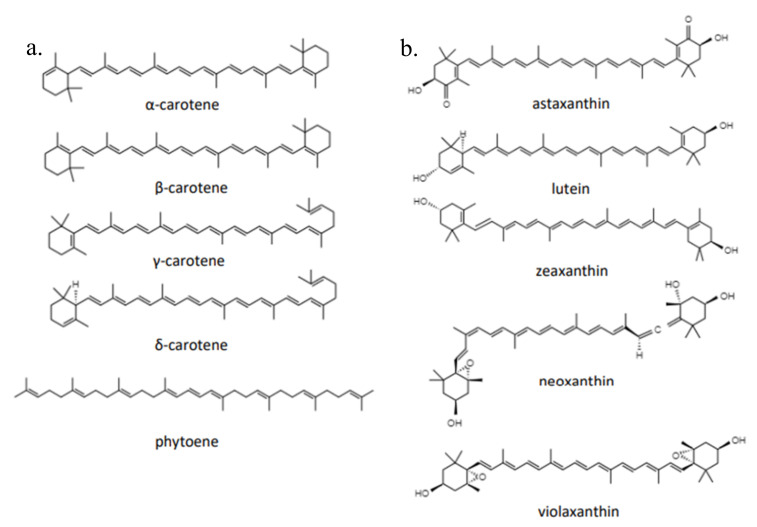
Various carotenoid structures. The basic structure of carotenoids is a tetraterpene. Carotenoid variations result from the presence of various side groups and different double-bond forms. (**a**) Carotene group; (**b**) xanthophyll group.

In general, carotenoids are categorized into carotenes in the form of unsaturated hydrocarbons (Figure 2), such as α-carotene, β-carotene, γ-carotene, δ-carotene, and phytoene as well as xanthophylls containing oxygen with substituents of hydroxyl groups, keto, epoxy, and aldehydes consisting of lutein, zeaxanthin, neoxanthin, violaxanthin, and astaxanthin [26]. α-carotene and β-carotene are isomers that have the same molecular formula (C_40_H_56_). Lycopene is a symmetrical tetraterpene that can undergo isomerization to any of a number of *cis*-isomers when exposed to heat [27]. Cyclization of lycopene by lycopene cyclase epsilon forms γ-carotene [28]. In nature, lutein (C_40_H_64_O_2_) and its stereoisomer zeaxanthin coexist with all *trans*-lutein as the most common geometric isomer [29]. When exposed to excess light, zeaxanthin itself can be formed from de-epoxidized violaxanthin [30]. Similar to lutein, neoxanthin (C_40_H_64_O_4_) is often present in all *trans*- or 9 *cis*-isomer forms [31]. Cryptoxanthin (C_40_H_64_O_4_) is an oxygenated carotenoid with one hydroxyl group molecule and is classified as a major xanthophyll carotenoid [32].

**Figure 3:**
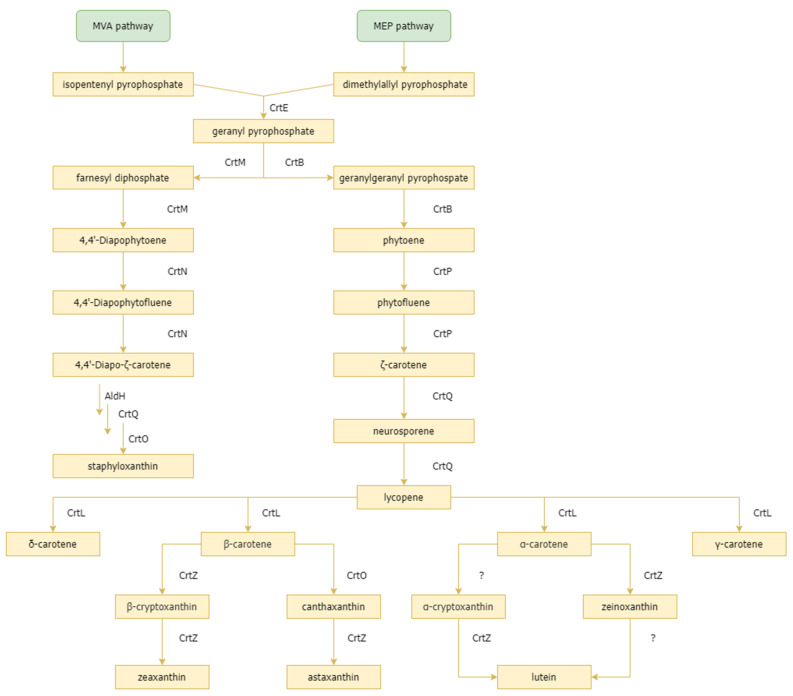
Carotenoid biosynthesis pathway. Various kinds of carotenoid derivatives are synthesized based on the genes contained in the carotenoid gene cluster. MVA and MEP pathways are shown in green. Precursor, intermediates, and final product compounds are shown in yellow. Enzymes that play a role in the conversion of compounds are written next to the metabolic pathways.

At least 1200 types of carotenoids have been discovered in 722 organisms [33]. Carotenoid pigments, which range in color from yellow to red, can be synthesized by plants, algae, fungi, and bacteria. Each organism can synthesize different types of carotenoids, depending on the precursors, metabolic pathways, gene clusters, and the needs of each species. As a terpene compound, the raw material for carotenoids is C5 in the form of isopentenyl pyrophosphate (IPP) and its isomer dimethylallyl pyrophosphate (DMAPP), which are synthesized from the mevalonate (MVA) and non-mevalonate (MEP) pathways [34,35]. The formation of geranyl pyrophosphate (GPP) and elongation of farnesyl diphosphate (FPP), which is then converted to geranylgeranyl pyrophosphate (GGPP), occurs as a result of head–tail condensation of IPP and DMAPP. Phytoene has the molecular formula C_40_H_64_ and is formed from two molecules of GGPP. Phytoene undergoes desaturation to form lycopene. Lycopene can further be converted to β-carotene via lycopene cyclase [36]. β-carotene hydroxylase will convert β-carotene into zeaxanthin, while if β-carotene ketolase and β-carotene hydroxylase are working, then β-carotene will be converted to astaxanthin [37]. The entirety of the carotenoid biosynthesis pathway can be visualized in Figure 3.

Under conditions of high environmental stress such as high salinity, high temperature, and nitrogen deficiency, the microalga *Haematococcus pluvialis* accumulates astaxanthin in an effort to protect and adapt [38]. Meanwhile, *Monascus purpureus* YY-1 upregulates carotenoid biosynthesis as a result of carbon deficiency [39]. Cyanobacteria, which are abundant in marine ecosystems, also produce the highest number of carotenoids in the form of carotene and a variety of xanthophylls, including caloxanthin, echinenone, synechoxanthin, canthaxanthin, myxoxanthophyll, nostoxanthin, and zeaxanthin [40]. Carotene, which is found in the cytoplasmic membrane and thylakoid cyanobacteria, acts as a protector by scavenging singlet oxygen. It is also present in photosystem II (PS II), where it forms complexes with proteins that aid in the photosynthesis process. Unlike carotenes, xanthophylls have not been clearly localized. However, it is known to have stronger activity than carotene [40].

In general, animals can only convert carotenoids to their derivatives, retinoids and apocarotenoids. However, some arthropods have been reported to be capable of producing carotenoids due to the presence of carotenoid synthesis genes [41]. Carotenes are critical for bacterial adaptation to their environment, including photoprotection and light harvesting. Polyene double bonds in carotenoids act as scavengers of free radicals [42] and as effective deactivators of excited molecules involved in radical formation, as well as singlet molecular oxygen, conferring anti-oxidant activity [43,44].

Carotenoids also act as accessory light-harvesting complexes that absorb light between 400 and 500 nm, protecting photosynthetic organisms from photodamage and photoquenching [45]. Light harvesting efficiency can also be achieved with the assistance of carotenoids in conditions of low light availability [46]. In contrast, when organisms are exposed to high-intensity light, carotenoids provide protection by scavenging reactive oxygen species (ROS) or non-photochemical quenching (NPQ) phenomena to reduce excess energy, which would impair photoinhibition in the photosystem [47]. Carotenoids protect cells from ultraviolet (UV) radiation and oxidative stress [48] and contribute to the fluidity of membranes [49]. The fluidity and structure of the cell membrane must be maintained in order for the cell to grow at low temperatures and regulate nutrient transport.

### 2.2. Carotenoid Synthesis Gene Cluster in Marine Microorganisms

Numerous genes are involved in carotenoids synthesis and interact with one another, forming a gene cluster. The carotene and other isoprenoid precursors, such as isopentenyl pyrophosphate (IPP) and its isomer dimethylallyl pyrophosphate (DMAPP), are formed through the mevalonate and non-mevalonate pathways [34,35]. The IPP isomerase, which is encoded by the *idi* gene, assists in switching between IPP and DMAPP. Other genes that play a role are *crtE*, which encodes GGPP synthase, *crtB*, which encodes phytoene synthase, *crtI*, which encodes phytoene desaturase, *crtY*, which encodes lycopene β-monocyclase, *crtW*, which encodes β-carotene ketolase, *crtX*, which encodes for zeaxanthin glycolases, *crtZ*, which encodes β-carotenoid hydroxylase, *crtZ*, which encodes desaturase, *crtQ*, which encodes ζ-carotene desaturase, *crtO*, which encodes β-carotene ketolase, *crtG*, which encodes astaxanthin hydroxylase, and *crtC*, *crtD*, and *crtF*, which play a role in spirilloxanthin biosynthesis [36,50,51].

**Figure 4:**
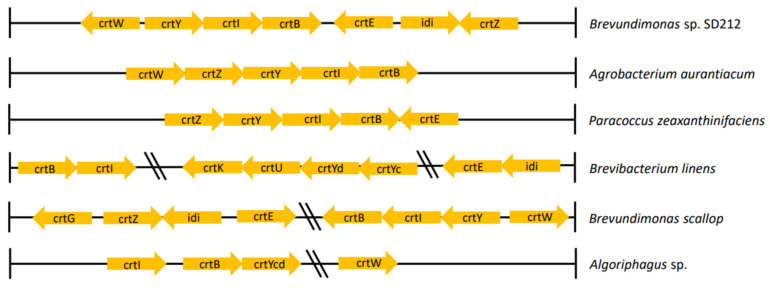
Variations in marine bacteria carotenoid gene clusters. *Brevundimonas* sp. carotenoid gene cluster consists of *crtW*, *crtY*, *crtI*, *crtB*, *crtE*, *idi*, and *crtZ* genes to synthesize 2-hydroxyastaxanthin; *Agrobacterium aurantiacum* consists of *crtW*, *crtZ*, *crtY*, *crtU*, and *crtB* genes; *Paracoccus zeaxanthinifaciens* consists of *crtZ*, *crtY*, *crtI*, *crtB*, and *crtE* genes; *Brevibacterium linens* consists of *crtB*, *crtI*, *crtK*, *crtU*, *crtYd*, *crtYc*, *crtE*, and *idi* genes; *Brevundimonas scallop* consists of *crtG*, *crtZ*, *idi*, *crtE*, *crtB*, *crtI*, *crtY*, and *crtW* genes; and *Algoriphagus* sp. consists of *crtI*, *crtB*, *crtYcd*, and *crtW* genes.

Gene clusters that play a role in the synthesis of carotenoids vary in each bacterial species, resulting in different carotenoid products. Several gene clusters involved in the biosynthesis of carotenoids in marine bacteria are highlighted in this review article (Figure 4). In the bacteria *Brevundimonas* sp. strain SD212, 2-hydroxyastaxanthin is synthesized with gene clusters, including *crtW*, *crtY*, *crtI*, *crtB*, *crtE*, *idi*, and *crtZ* [52]. Liu et al. [37] demonstrated that a gene cluster composed of *crtE*, *crtB*, *crtI*, *crtY*, *crtW* + *crtZ*, and *crtG* from the bacterium *Brevundimonas scallop* isolated from the noble scallop *Chlamys nobilis* produces the final product, 2,2’-dihydroxy-astaxanthin. Meanwhile, in the marine bacterium *Agrobacterium aurantiacum* that produces astaxanthin, the gene clusters that play a role include *crtW*, *crtZ*, *crtY*, *crtI*, and *crtB* [53,54]. There are some differences in the structure of the gene clusters of the three bacteria that produce the carotenoid astaxanthin. Gram-negative bacteria *Paracoccus zeaxanthinifaciens* previously identified as *Flavobacterium* sp. strain R1534 can synthesize zeaxanthin with carotenoid gene clusters found in its genome, including the *crtE*, *crtB*, *crtY*, *crtI*, and *crtZ* genes [55]. Meanwhile, the carotenoid gene cluster found in *Brevibacterium linens* consists of *crtB*, *crtI*, *crtK*, *crtU*, *crtYd*, *crtY*, *crtE*, and *idi* to produce the final product, β-carotene derivative in the form of isorenieratene [56]. Flexixanthin, synthesized by *Algoriphagus* sp., cannot be separated from the role of the gene cluster consisting of *crtI*, *crtB*, *crtY*, and *crtW* [57].

## 3. Marine-Derived Carotenoid Screening

### 3.1. In Vitro Carotenoid Screening

#### 3.1.1. Culture-Dependent Approaches

Microbial screening refers to the detection and isolation of microorganisms present in a certain sample. Culture-dependent methods in the analysis of microbial communities for further screening purposes, such as identification of specific secondary metabolic properties, rely on the in vitro cultivation of microorganisms obtained from a certain sample [58]. Owing to the immense diversity within the physiological properties of microorganisms, various types of media have been developed in order to support the growth of different microorganisms—which thrive in different natural environments—under laboratory conditions.

Kusmita et al. [59] described one method for screening marine-derived carotenoid-producing microorganisms, which involves serial dilution of bacterial samples and 48 h incubation of said samples at 30 °C in a dish containing seawater yeast peptone agar [59]. In general, culture-dependent screening methods provide several advantages: (1) the ability to assess living microorganisms, (2) the ability to screen viable cells within a sample, and (3) the high sensitivity demonstrated by microorganisms when cultured within appropriate growth media [60]. Despite these advantages, culture-dependent methods would still be insufficient in representing the entirety of the microbial community present in a marine-derived sample, as marine microorganisms have been regarded as notoriously hard to cultivate in vitro [61].

Numerous obstacles exist when it comes to cultivating marine microbial communities in the laboratory: (1) the imbalance of growth between different microbial species within a community when they are removed from their natural environment, (2) the faulty cellular processes that occur within these microorganisms when placed under laboratory conditions, (3) the probability of viral invasion, which may inhibit bacterial growth, (4) the incompatibility between microorganisms and the substrates present in culture media, and (5) the toxicity induced by high concentrations of media required for detectable growth [61]. Furthermore, even in culturable marine microorganisms, secondary metabolite production may be hindered by the axenic conditions associated with in vitro culturing methods [58]. In general, these factors render culture-dependent methods incapable of accurately portraying a microbial community.

#### 3.1.2. Culture-Independent Approaches

Culture-independent microbial screening methods involve directly extracting and sequencing microbial genetic material present in a sample without the need for cultivation. A common example of this is metabarcoding, which involves the amplification and sequencing of genetic barcodes—specifically the 16S rRNA gene in prokaryotes—directly from a sample. The sequence data may be analyzed for further screening purposes. This particular method provides several advantages: (1) the ability to detect species missed by culture-dependent methods, (2) the high specificity involved in further screening, (3) the straightforward and relatively rapid processing involved, and (4) the option to freeze samples for later analysis [62]. However, PCR-based methods intrinsically present an amplification bias. This bias could be overcome by employing an alternative culture-independent approach: shotgun metagenomics, which involves the indiscriminate sequencing of all microbial genomes present in a sample without previous amplification [63].

Shotgun metagenomics has generally been considered a viable method for conducting large-scale analyses of complex microbiota [63]. However, this method is not without limitations, as shotgun metagenomic analysis necessitates additional processing to overcome host or non-target DNA dominance [58]. In general, the limitations and advantages of culture-dependent and culture-independent methods may complement one another in terms of providing an optimal and accurate description of microbial communities during secondary metabolite production screening. These combined methods can be used in screening carotenoid biosynthetic gene clusters that are hidden within a variety of marine bacteria.

### 3.2. In Silico Carotenoid Screening

The secondary metabolite Biosynthetic Gene Clusters (smBGCs) are a group of genes that regulate a certain metabolic network to produce secondary metabolites. These genes perform a variety of tasks, including controlling expression, aiding biosynthesis of precursor molecules, assembling and modifying translation and post-translational products, and regulating product resistance. Abiotic stress and biotic variables found in the environment where the bacteria originate affect the control of the smBGCs network [64]. The disparity in environmental conditions between the laboratory and the natural ecosystem creates a barrier to the laboratory production of secondary metabolites. Under laboratory circumstances, some of these microbes’ genes are not expressed or are “hidden” or “cryptic”. The physical, chemical, and biological conditions for expression of the gene were not met. It is difficult to determine the existence of this gene using only laboratory detection methods. As a result, a strategy is required that considers the target microorganism’s metabolic capacity as a whole.

**Figure 5:**
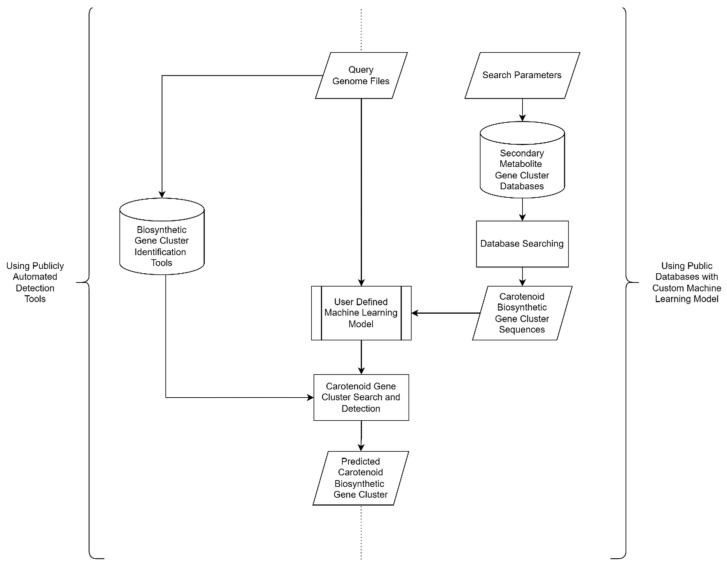
General workflow of in silico carotenoid screening using databases and tools.

Genome mining is one of the most frequently used techniques for resolving this issue. It is a method for identifying previously unidentified biosynthetic gene clusters. In general, genome mining can be divided into in silico and in vitro stages. Biosynthetic Gene Cluster (BGC) identification and characterization are among the in silico processes. This stage entails performing a variety of bioinformatics analyses on the genome sequences of the target microorganisms. Widely available databases and tools offer various utilities that can aid in silico screening for certain compounds. In general, the workflow of in silico natural product screening using various publicly available tools and databases are visualized in Figure 5, whereas the in vitro stage involves the identification and characterization of the BGCs product using wet laboratory protocols. When gene sequences are expected to encode specific genes, they will be expressed in certain vectors and characterized [64].

Rule-based approaches and machine learning-based methods are the two types of in silico secondary metabolite screening methods. The rule-based method utilizes a sequence alignment-based profile in the Hidden Markov Model (HMM) of smBGCs that belongs to a certain biosynthetic pathway and is available in the database [64]. Apparently, available tools and databases commonly encompass various natural products or secondary metabolites (Table 1). There are no specific tools and databases for carotenoid available currently. Thus, screening for a specific set of natural products such as carotenoids can potentially be conducted by changing search parameters in respective databases or tools.

Furthermore, various tools are needed for constructing HMM profiles and for applying other bioinformatics methods. Rapid and easy secondary metabolite screening can be performed using publicly available tools (Table 2) with genome files as the query. PRISM [65] and antiSMASH [66] are two tools that are often used to look for smBGCs using a rule-based strategy. By utilizing these rule-based methods, it is possible to efficiently search databases for hidden smBGCs that are similar. However, the disadvantage of this strategy is that it only finds smBGCs that are identical to those in databases, making it difficult to predict novel smBGCs that are not identical to those in databases.

Custom machine learning models and algorithms can be used in a more advanced screening pipeline, utilizing the biosynthesis gene cluster sequences available in the public databases (Table 1) as the training data set. For the discovery of novel smBGCs, an approach using machine learning-based methods such as ClusterFinder [67] and DeepBGC [68] is required. However, the drawback of this machine learning-based method is the high false-positive rate [69]. Nevertheless, the combination of these two approaches is expected to provide an accurate and representative list of smBGCs.

Previously, genome-based in silico screening has been used to identify two missing genes in the carotenoid biosynthesis pathway. BLAST+ was used to perform similarity searching against the local BLAST Protein database constructed using the makeblastdb program [64]. There was also research conducted using in silico genome-based screening to characterize marine bacterial carotenoid gene clusters using tools and databases as described in Table 1 [70,71,72,73,74,75,76]. The most commonly used tool in these research studies is the antiSMASH pipeline. Overall, these “genome mining” techniques are used to identify gene clusters, predicting their similarity with available gene clusters in the databases, and constructing a putative pathway of carotenoid biosynthesis.

## 4. Industrial Production of Carotenoid

### 4.1. Economic Potential of Marine Carotenoids

The industry for the utilization of carotenoid pigments has accelerated its growth in recent years. The global carotenoid market is being propelled forward by the increasing use of pigments in a variety of industries, including health and pharmaceutical [77]. Between 2016 and 2019, the market for carotenoid pigments surpassed 1.5 billion USD [78,79,80]. The market value of carotenoids is expected to reach 1.16 billion USD by the end of 2025, with a compound annual growth rate (CAGR) of 0.6% during 2019–2025 [81]. Carotenoid pigments, such as astaxanthin, carotene, and lutein, have a higher demand and account for 60% of the market compared to other pigments. This is due to the third pigment’s potential in the sectors of nutraceuticals, pharmaceuticals, food, animal feed, supplements, and cosmetics [82].

The astaxanthin market is the largest of all carotenoid compounds. The global astaxanthin market was worth 647 million USD in 2021 and is expected to reach 965 million USD in 2026, with a CAGR of 8.3% [83]. The astaxanthin market is expanding as the feed and cosmetics industries develop. Astaxanthin is widely used in the aquaculture industry as a salmon feed additive and as an antioxidant in the cosmetics industry [82,83]. Carotenoid pigments are currently in high demand in the livestock and aquaculture feed industries, as well as in dietary supplements. The demand for high-quality, natural carotenoid-based supplements will continue to grow in the future [83,84].

European countries have the largest carotenoid market when compared to the other regions of the world, and this is likely to account for a sizable portion of the worldwide market. The market for carotenoids in European countries was approximately 598.6 million USD in 2019, with a compounded annual growth rate (CAGR) of 3.2%. The enormous animal feed and cosmetics industries, as well as the food coloring industry, all contribute to the carotenoid market’s growth in Europe. This demonstrates that the development of carotenoids will not halt in the absence of industrial advances that utilize carotenoids.

### 4.2. Carotenoids Function and Uses in Health and Pharmaceutical Industry

Carotenoids have been widely used in the health industry. Photosynthetic organisms mainly produce carotenoids, which have the primary purpose of capturing light for photosynthesis purposes. Carotenoids, however, have a variety of secondary activities, one of which is to safeguard (photoprotect) photosynthetic organs against radiation damage. This is known as antioxidant activity, and it involves scavenging reactive oxygen species (ROS) such as the hydroxyl radical and hydrogen peroxide. This is the most commonly used biological function in human health [85,86]. The chemical structure and interaction of substances with biological membranes in organisms provide antioxidant activity. Astaxanthin, fukoxanthin, zeaxanthin, sioxanthin, saproxanthin, myoxol, siphoxanthin, and other carotenoid chemicals originating from the ocean have a significant potential to play a role in human health [86].

Astaxanthin produced by marine microalgae has antioxidant activity with various mechanisms, such as inhibiting the production of proinflammatory cytokines (nuclear factor kB, IL-1, IL-6, and TNF-), inhibiting the activity of the renin–angiotensin system, inhibiting the production of TGF-B1, and inhibiting the effect of antimicrobials in the body. Astaxanthin has enzyme targets within the cell in the form of superoxide dismutase (SOD) and catalase (CAT). Both enzymes play a role in reducing radical compounds in the body; SOD converts oxygen radicals (O_2-_) to oxygen, and CAT breaks down H_2_O_2_ into H_2_O and O_2_, reducing the accumulation of harmful radical compounds [87,88]. Astaxanthin is currently believed to have the potential to lower cancer risk and slow its progression. Astaxanthin has an anticancer impact on transplantable methylcholanthrene-induced fibrosarcoma (Meth-A tumor) cells, according to Jyonouchi et al. [89].

Atherosclerosis is one of the diseases that is mediated or influenced by free radicals. Damage to the endothelium and the accumulation of low-density lipoprotein cholesterol (LDL-C) in the arterial walls are the main causes of atherosclerosis, although the inflammatory process is also involved. Inflammation, endothelial dysfunction, and vascular remodeling are all part of the pathophysiology of atherosclerosis. Nitric oxide (NO) inactivation and vascular NO bioavailability are linked to increased ROS. Astaxanthin has been demonstrated to improve endothelial function in resistant arteries, reduce oxidative stress, and increase NO bioavailability. Anti-obesity and immune system stimulation are two further benefits of astaxanthin for health [87,90].

Fucoxanthin is a xanthophyll found in brown seaweeds (Phaeophyceae) such as *Undaria pinnatifida, Hijikia fusiformis, Laminaria japonica*, and *Sargassum fulvellum*. Because of its molecular structure, which includes allenic bonds, epoxy groups, and conjugated carbonyl groups in the polyene backbone, this pigment is known for its high antioxidant activity. Fucoxanthin were found to be effective in reducing risk factors for cardiovascular disease, including obesity, diabetes, hypertension, chronic inflammation, plasma levels, hepatic triglyceride levels, and cholesterol concentrations [86].

Fucoxanthin has been shown to have antiproliferative properties, suggesting that it could be used to treat cancer. Fucoxanthin induces the apoptotic cascade by promoting caspase cleavage and lowering *Bcl-xl* gene expression. PC-3 proliferation is inhibited by one of fucoxanthin’s metabolites, fucoxanthinol. Halocynthiaxanthin, found in Halocynthia roretzi, is a derivative metabolite of fucoxanthin. After having been proven to have a larger cytotoxic effect on human neuroblastoma cells (GOTO) than fucoxanthin, this metabolite has the potential to be used as an anticancer agent [87,91].

One of the types of fucoxanthin metabolite is mytiloxanthin; this pigment is found in tunicates and shellfish. The antioxidant activity of this metabolite is higher than that of astaxanthin. This is because mytiloxanthin has an 11-conjugated-double-bond polyene system, as well as carbonyl and hydroxyl groups, in its structure. When compared to other pigments, this structure enhances the pigment’s inhibitory effect on lipid peroxidation [87,92].

Zeaxanthin is a non-provitamin A oxygenated carotenoid that complements lutein chemically. The marine bacterium *Gramella oceani* of the Flavobacteriaceae family provides zeaxanthin. The retinal portion of the eye, specifically the macula lutea, is where zeaxanthin is found. Zeaxanthin levels in these areas are directly proportional to the thickness of the augmented macular pigment, which can reduce the risk of age-related macular degeneration (AMD). This is due to the antioxidant activity of zeaxanthin and its capacity to act as a blue light filter that protects retinal tissue from damage [93]. Zeaxanthin also protects proteins, lipids, and DNA from harm via modulating antioxidant systems such intracellular GSH [86,93].

Rare carotenoid molecules such as siphonaxanthin, saproxanthin, myoxol, and sioxanthin offer a lot of untapped promise in terms of health applications. Green algae from the sea, such as *Codium fragile, Codium cylindricum, Caulerpa lentilifera*, and *Umbraulva japonica*, produce the siphoxanthin pigment. This pigment has a pro-apoptotic effect and can effectively stop human leukemia HL-60 from spreading. A strong inhibitory effect is observed at the molecular level, this pigment plays a role in caspase-3 stimulation and upregulation of death receptor 5 (DR5) protein expression. *Bcl-2* and *GADD45* are two more genes that have been targeted, suggesting that the antiproliferative effect is mediated by pigment-specific activation of apoptotic pathways. Siphonaxanthin can also decrease the glycation process toward the end of the inflammatory reaction and modify the inflammatory response. This pigment prevented the buildup of lipids in experimental rats in vitro. In 3T3-L1 cells and hepatocytes, siphonaxanthin has been shown to inhibit lipogenesis [86,87,94,95].

Saproxanthin and myoxol are monocyclic carotenoids with a γ-carotene skeleton that are widely found in bacteria from the Flavobacteriaceae family. In neural hybridoma cell lines, both of these drugs displayed inhibitory activity against lipid peroxidation in the brain, antioxidant activity, and neuroprotective activity against L-glutamate toxicity. The hydroxyl groups at the acyclic terminals play a structural role in the strengthening and stabilization of biological membranes, lowering their oxygen permeability. They have stronger antioxidant activity than zeaxanthin and β-carotene [87,96].

### 4.3. Production System and Mechanism in Industrial Scale

On an industrial scale, there are three different methods of producing carotenes. The first is a physicochemical method, which is the more traditional method of production that involves extracting carotene-containing plant material. Carotenoids are commercially extracted from green plant parts such as roots, flowers, and fruits. Additionally, these pigments are found in vegetable crops such as carrots and tomatoes. Carotene is located in the thylakoid membrane, which is an organelle in chloroplasts. The primary disadvantage of this technology is its high production cost, which is geographically and seasonally dependent on obtaining high-quality raw materials.

Chemical synthesis is the second method. Since 1950, researchers have been attempting to chemically synthesize carotenoids [97]. Synthetic carotenoids are now widely used as colors and feed additives. Lycopene, canthaxanthin, astaxanthin, β-carotene, and citranaxathin are the carotenes that have been effectively manufactured on an industrial scale [97].

**Figure 6:**
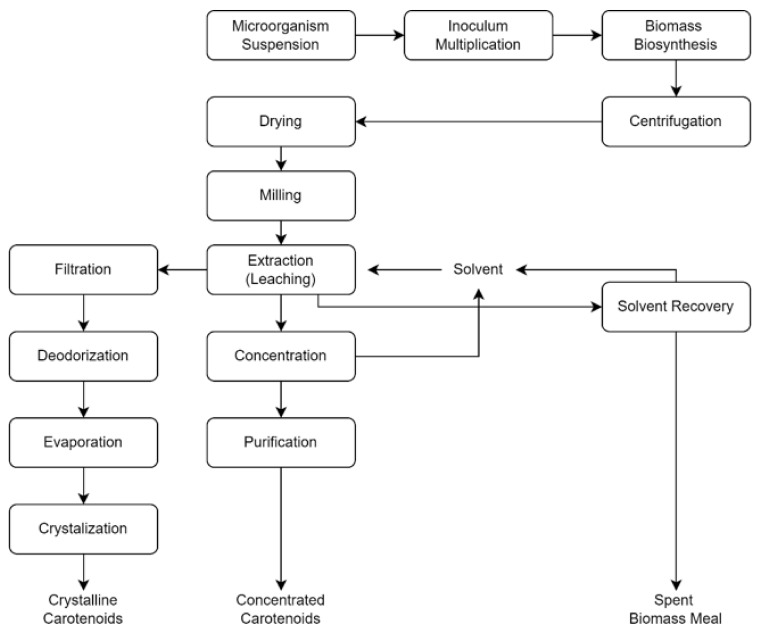
Main steps in the microbiological biosynthesis route for carotenoid production.

The third method, known as microbiological biosynthesis. This method takes advantage of microorganisms that can produce carotene naturally. Those microorganisms will be grown and chemically extracted to produce the required pigments. The production process of carotenoids in industry are summarized [97,98,99] and visualized in Figure 6. This process has several advantages that compensate for the disadvantages of traditional methods, including low production costs, greater control, a shorter manufacturing time, and a natural end product. Because the use of synthetic carotene is currently restricted in the industrial sector, this approach is regarded as the best choice to replace it [97,100].

Increasing the efficiency of carotene biosynthesis is one strategy to boost carotene output with this method. The amount of enzyme and its activity level, as well as the amount of carbon or precursors accessible in the external environment, influence carotene biosynthesis [101]. Carotene production in microbes can be increased by providing stimulants and optimizing environmental conditions during the cultivation process. The addition of mevalonic acid as a precursor was found to increase carotene formation by 120% in *R. mucilaginosa* and 35% in *R. glutinis* [102].

Currently, there are three biosynthetic approaches that can be used to increase carotenoid production, including pathway engineering (metabolic engineering), host genetic modification, and protein engineering for enzyme and pathway optimization [103]. Metabolic engineering is the alteration of metabolic pathways with genetic modifications, such as the introduction of new genes with recombinant DNA technology. The goal of metabolic engineering is to employ molecular biology tools to conduct a systematic investigation of metabolic pathways in order to optimize cell properties through rational genetic modification [72].

Insertion of the *crtE* gene from the bacteria *P. agglomerans*, chromosome integration of the MVA pathway, and insertion of the *idi* gene into the *E. coli* chromosome can increase the production of carotenoids (lutein) [104]. To increase the production yield, it is also necessary to consider the key enzymes, which are the limiting factors in the production of carotenoids. Enzymes with enhanced activity and improved gene expression associated with carotenoid synthesis are necessary for future improvements. For example, in the production of β-carotene, the enzyme resulting from the translation of the *crtYB* gene becomes a limiting enzyme; thus, it must be produced in higher amounts than other enzymes to obtain higher carotene production [105].

Carotenoid production via operons could also be increased by changing the bioparts i.e., regulatory bioparts used in the genetic circuit [106]. These two concepts create a gap in the need for bioparts that can increase the expression of carotenoid-producing genes and produce higher enzyme activity. This can be achieved by creating a new synthetic system composed of optimized bioparts with a high level of efficiency. Based on the results of research by Dou et al., [107] the strong promoters of the fungus *Pichia pastoris*, namely PGAP, PGCW14, P0208, P0019, P0230, and P0107, have been able to increase the expression of β-carotene genes, thereby increasing the amount of β-carotene production. The expression of β-carotene on the chassis in the form of *P. pastoris* has a higher yield than *S. cerevisiae*, such that *P. pastoris* can be a new chassis for beta-carotene production [105].

Currently, there are two operons that can be formed to increase carotenoid production, namely operons related to genes in the carotene pathway and operons for genes related to the MEP and MVA pathways. The production of carotenoids in the form of zeaxanthin can be increased by 4.4 times with the formation of a synthetic operon for the carotenoid-producing gene (*crt*), namely *crtE*-*crtB*-*crtI*-*crtY*-*crtZ* in *E. coli* with gene sequences on the operon according to the zeaxanthin biosynthetic pathway [108]. Production with a high yield requires a high supply of precursors and an optimal production line [109]. The combination of the plasmid pHCMC04G with the xylose inducible promoter and eight MEP pathway-related genes, namely *dxs*, *ispD*, *ispH*, *ispF*, *ispC*, *ispE*, *ispG*, and *ispA*, into a single operon with a similar sequence resulted in the production of carotenoids up to 20 times that of the control [110].

## 5. Conclusions

Carotenoids are in high demand due to their numerous applications, including antioxidant, anticancer, and anti-obesity. Numerous marine bacteria species are capable of synthesizing a variety of carotenoids, depending on the carotenoid synthesis gene cluster present in their genome. Advances in genome sequencing and bioinformatics, combined with developed tools, algorithms, and databases for genome mining, may aid in the discovery of secondary metabolite gene clusters in a variety of marine-derived microorganisms. A combination of in silico and in vitro approaches could potentially increase the rate of discovery of novel secondary metabolites such as carotenoids. Furthermore, recent advances in biotechnological industry upscaling and newly emerging production methods would assist industries in meeting this ever-increasing demand.

## Figures and Tables

**Table 1:** Currently available databases for screening secondary metabolites.

No.	Name	Database URL
1	antiSMASH database	http://antismash-db.secondarymetabolites.org/
2	Bactibase	http://bactibase.pfba-lab-tun.org
3	ClusterMine360	http://www.clustermine360.ca/
4	ClustScan Database	http://csdb.bioserv.pbf.hr/csdb/ClustScanWeb.html
5	DoBISCUIT	http://www.bio.nite.go.jp/pks/
6	IMG-ABC	https://img.jgi.doe.gov/abc
7	MIBiG	https://mibig.secondarymetabolites.org/

**Table 2:** General BGC identification tools.

No.	Tool	Web URL
1	antiSMASH 6.0	http://antismash.secondarymetabolites.org/
2	Artemis	http://www.sanger.ac.uk/science/tools/artemis
3	ClusterFinder	http://github.com/petercim/ClusterFinder
4	ClusterMine 360	http://clustermine360.ca/
5	eSNaPD	http://esnapd2.rockefeller.edu/
6	FramePlot 4.0beta	http://nocardia.nih.go.jp/fp4
7	IMG-ABC	https://img.jgi.doe.gov/cgi-bin/abc/main.cgi
8	MultiGeneBlast	http://multigeneblast.sourceforge.net/
9	NP.Searcher	http://dna.sherman.lsi.umich.edu/
10	NaPDoS	http://napdos.ucsd.edu/
11	SMURF	https://www.jcvi.org/smurf
12	HMMER	http://www.ebi.ac.uk/Tools/hmmer/search/jackhmmer

## Data Availability

Not applicable.
